# Genetic variations that influence paclitaxel pharmacokinetics and intracellular effects that may contribute to chemotherapy-induced neuropathy: A narrative review

**DOI:** 10.3389/fpain.2023.1139883

**Published:** 2023-05-12

**Authors:** Ken B. Johnson, Anukriti Sharma, N. Lynn Henry, Mei Wei, Bihua Bie, Courtney E. Hershberger, Emily E. Rhoades, Alper Sen, Ryan E. Johnson, Jacob Steenblik, Jennifer Hockings, G. Thomas Budd, Charis Eng, Joseph Foss, Daniel M. Rotroff

**Affiliations:** ^1^ Department of Anesthesiology, University of Utah, Salt Lake City, UT, United States; ^2^ Department of Quantitative Health Sciences, Lerner Research Institute, Cleveland Clinic, Cleveland, OH, United States; ^3^ Department of Internal Medicine, University of Michigan Medical School, Ann Arbor, MI, United States; ^4^ Huntsman Cancer Institute, University of Utah, Salt Lake City, UT, United States; ^5^ Department of Anesthesiology, Cleveland Clinic, Cleveland, OH, United States; ^6^ Taussig Cancer Institute, Cleveland Clinic, Cleveland, OH, United States; ^7^ Genomic Medicine Institute, Lerner Research Institute, Cleveland Clinic, Cleveland, OH, United States; ^8^ Department of Pharmacy, Cleveland Clinic, Cleveland, OH, United States; ^9^ Endocrinology and Metabolism Institute, Cleveland Clinic, Cleveland, OH, United States; ^10^ Center for Quantitative Metabolic Research, Cleveland Clinic, Cleveland, OH, United States

**Keywords:** paclitaxel, pharmacokinetics, chemotherapy-induced neuropathy, genetic, epigenetic

## Abstract

Taxanes, particularly paclitaxel and docetaxel, are chemotherapeutic agents commonly used to treat breast cancers. A frequent side effect is chemotherapy-induced peripheral neuropathy (CIPN) that occurs in up to 70% of all treated patients and impacts the quality of life during and after treatment. CIPN presents as glove and stocking sensory deficits and diminished motor and autonomic function. Nerves with longer axons are at higher risk of developing CIPN. The causes of CIPN are multifactorial and poorly understood, limiting treatment options. Pathophysiologic mechanisms can include: (i) disruptions of mitochondrial and intracellular microtubule functions, (ii) disruption of axon morphology, and (iii) activation of microglial and other immune cell responses, among others. Recent work has explored the contribution of genetic variation and selected epigenetic changes in response to taxanes for any insights into their relation to pathophysiologic mechanisms of CIPN20, with the hope of identifying predictive and targetable biomarkers. Although promising, many genetic studies of CIPN are inconsistent making it difficult to develop reliable biomarkers of CIPN. The aims of this narrative review are to *benchmark* available evidence and *identify gap*s in the understanding of the role genetic variation has in influencing paclitaxel's pharmacokinetics and cellular membrane transport potentially related to the development of CIPN.

## Introduction

A common side effect of taxanes, chemotherapeutic used to treat breast, ovarian, and many other solid tumors, is chemotherapy-induced peripheral neuropathy (CIPN). CIPN can be severe enough to disrupt treatment and produce significant patient morbidity as well as long lasting effects even after remission of disease. Researchers are highly motivated to discover new ways to identify patients at risk for developing treatment limiting CIPN before therapy is initiated so that dose adjustments can be made to ensure treatment completion.

**Figure 1:**
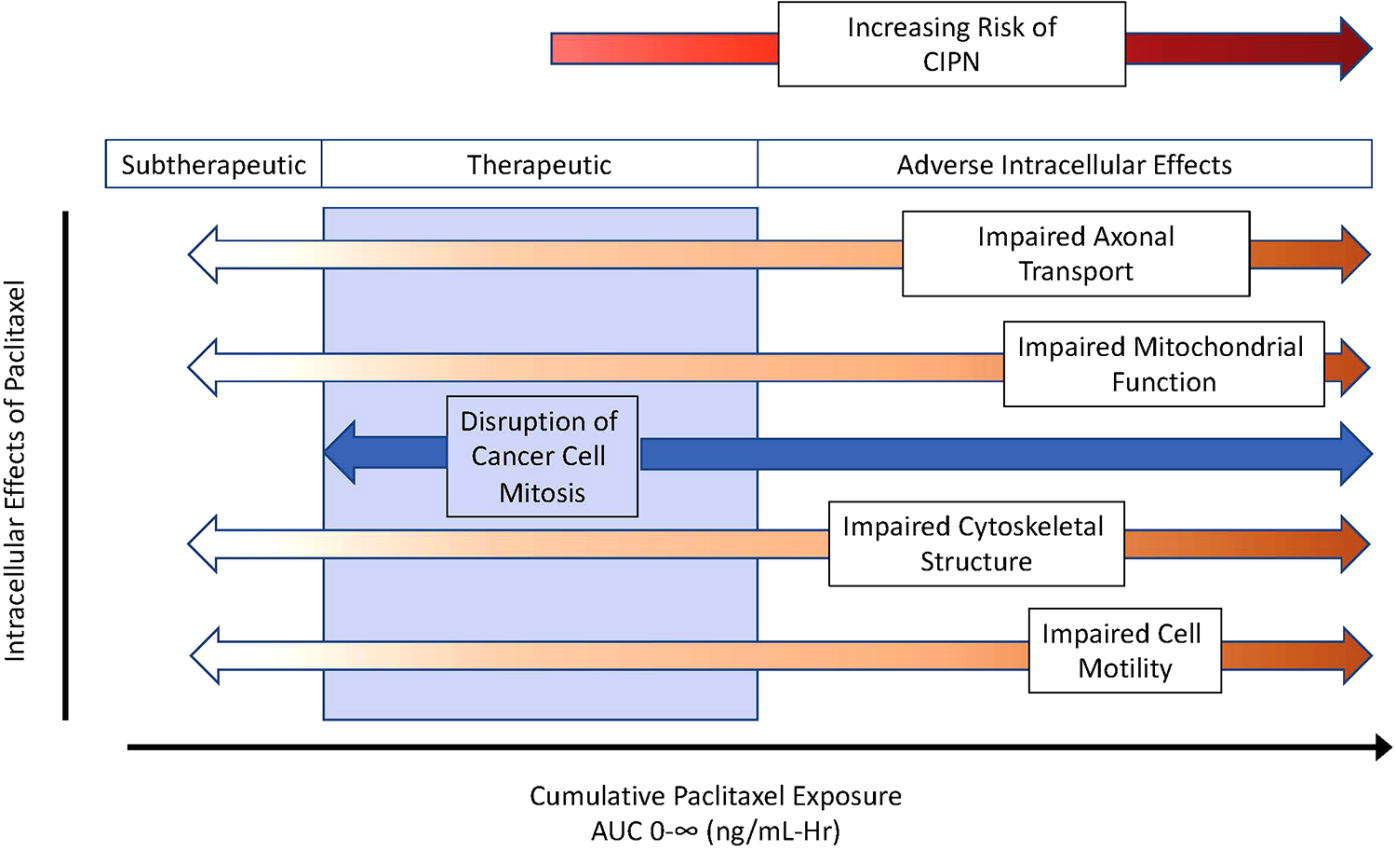
Theoretical considerations of desired and adverse paclitaxel effects across exposure, as described using area under the curve for plasma concentrations over time. As cumulative exposure increases, paclitaxel effectiveness transitions from subtherapeutic to therapeutic (blue zone) and then to toxicity manifest by adverse intracellular effects increasing the risk of chemotherapy induce peripheral neuropathy (CIPN). AUC 0-∞ indicates the area under the plasma concentration over time curve out to infinity.

Paclitaxel's predominant antineoplastic mechanism of action involves binding to beta subunits of tubulin that make up microtubules. Once bound, paclitaxel stabilizes microtubules, disrupting intracellular functions including the separation of chromosomes during cell mitosis, intracellular transport, and cell motility, among others. What remains poorly defined is the optimal paclitaxel exposure adequate to disrupt cancer cell growth yet minimize adverse consequences of impaired microtubule function in healthy cells. Some researchers argue that signs of toxicity also represent signs of efficacy, but the challenge is to find a dose that minimizes dose-limiting toxicity yet maximizes effectiveness. Figure 1 presents a schematic of theoretical considerations of desired and adverse effects following paclitaxel exposure. There is likely no clean break between therapeutic and toxic exposures, but some overlap exists, and the extent of this overlap varies between patients. In this hypothetical schematic, a few patients may develop CIPN even at subtherapeutic exposures. Most patients may develop CIPN at therapeutic exposures, but some will not. As exposure increases, the desired anticancer effects persist but with an increasing prevalence of neurotoxicity. With a narrow, or perhaps nonexistent therapeutic index, understanding sources of interindividual paclitaxel pharmacokinetic variability is of keen interest.

Our group is conducting a multicenter prospective observational study measuring biomarkers that may predict the development of CIPN from genetic, epigenetic, metabolomic, demographic and clinical domains before, during, and after taxane therapy for breast cancer (1). The rationale for this work is that selected biomarkers alone or in combination may guide personalized taxane dosing that achieves therapeutic goals while minimizing adverse consequences of CIPN. The scope of our study is to collect biomarkers over 12 months in 400 patients treated with taxanes for non-metastatic breast cancer. Of particular interest is a better understanding of how genetic and other molecular biomarkers, in combination with other predisposing factors such as diet, lifestyle and clinical history, may contribute to identifying patients at risk for CIPN. Given that previous genomic studies have not yielded consistent results, we want to determine not only how selected gene variants present *before* taxane therapy identify patients at risk for developing CIPN, but also how other molecular biomarkers such as mRNA expression, micro RNAs, and DNA methylation, along with other transcription factors, measured *during* taxane therapy may identify patients at risk for CIPN that were not otherwise identified before taxane therapy was started.

The focus of this narrative review is to benchmark the physiologic and pharmacologic features of genetic variation that may serve as useful biomarkers in identifying those at risk of developing CIPN when treated with *paclitaxel*. Likely pathophysiologic contributors to CIPN include (i) variation in drug metabolism, (ii) predisposing genetic factors to neuropathic pain susceptibility, (iii) neuropsychological contributions such as depression & catastrophizing, and (iv) environmental exposures and diet. Although mechanisms of CIPN are multifactorial, including adverse intracellular effects such as impaired cytoskeletal structure, mitochondrial function, axonal transport, and impaired cell motility, we have focused this narrative review specifically on the contributions of variations in paclitaxel pharmacokinetics and cellular membrane transport on CIPN. We have summarized associations between kinetic parameters or variants of genes that are responsible for metabolizing paclitaxel and what is known about their relationship to CIPN development. Additional details about the clinical annotations for gene variant relationships with paclitaxel can be found on the PharmGKB website (https://www.pharmgkb.org/chemical/PA450761/clinicalAnnotation). Our aim was to better understand what is known and what remains poorly understood regarding paclitaxel exposure from conventional dosing regimens used to treat breast cancer.

## Paclitaxel pharmacokinetic parameters and CIPN

**Figure 2:**
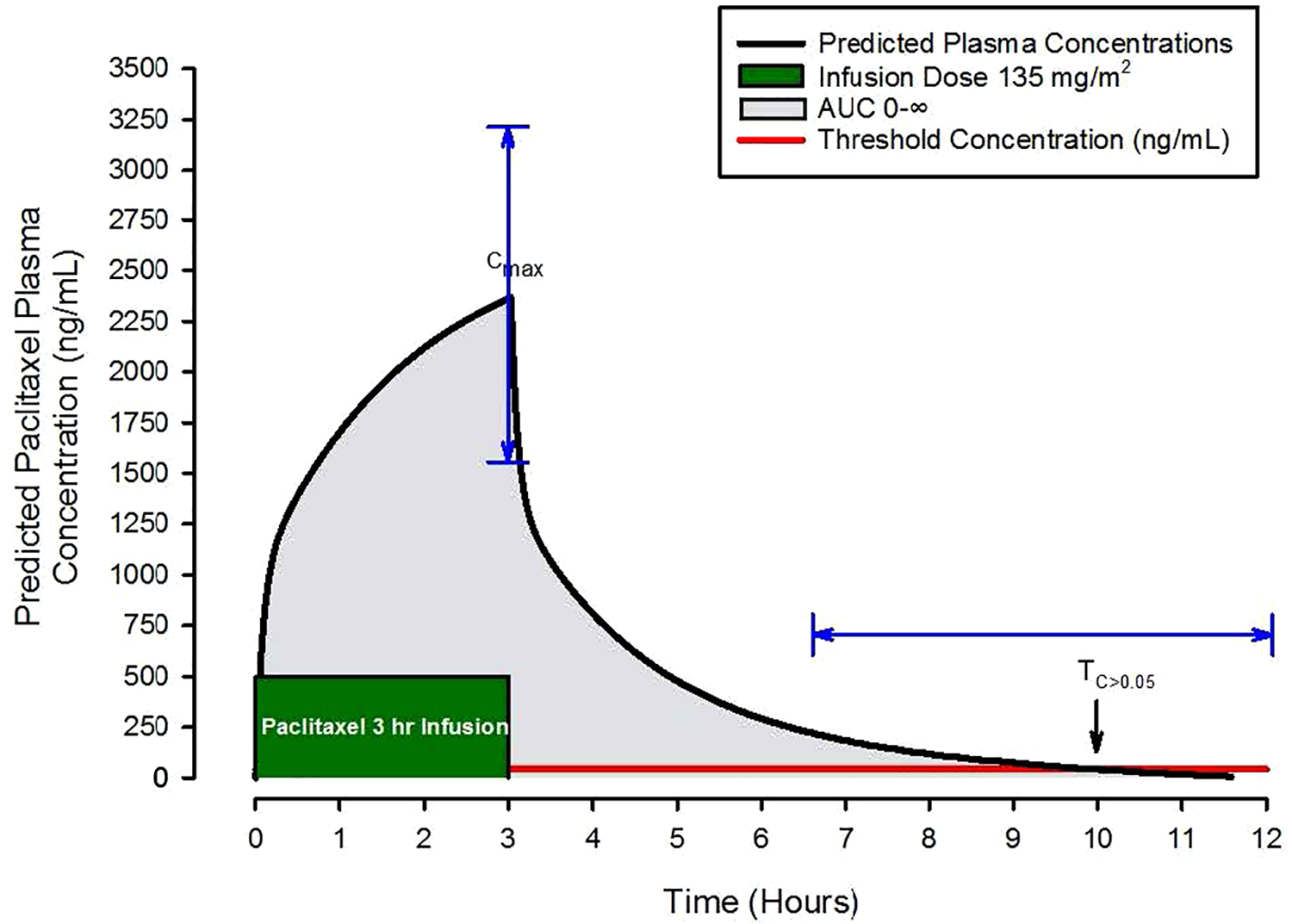
Schematic of predicted paclitaxel plasma concentrations following a three-hour paclitaxel infusion of 135 mg/m^2^. Pharmacokinetic parameters of interest include the maximal concentration (Cmax), the area under the plasma concentration over time curve out to infinity (AUC 0-∞), grey shaded area, and the time above an estimated toxicity threshold of 0.05 µmol/L (43 ng/ml) for CIPN abbreviated as T_C>0.05_, the red horizontal line. The blue arrows demonstrate the magnitude of variability in Cmax and T_C>0.05_.

For breast cancer, paclitaxel is primarily administered as 1- or 3-hour continuous intravenous infusions. Investigators have described paclitaxel kinetics by frequently measuring plasma concentrations before, during, and after infusions and using this data to create kinetic models (2, 3). As an example, consider the Figure 2 schematic presentation of model predictions of paclitaxel concentrations over time following a 3-hour infusion. A few important features of paclitaxel's pharmacologic behavior as it relates to drug exposure are easily visualized. These include the maximal concentration (Cmax), area under the plasma concentration over time curve out to infinity (AUC 0-∞), and the time above an estimated toxicity threshold of 0.05 µmol/L (43 ng/ml) for CIPN abbreviated as T_C>0.05_. For example, with Cmax, plasma concentrations above 2,885 ng/ml or T_C>0.05_ > 14 h are associated with an increased risk of treatment limiting CIPN (4). Cmax, T_C>0.05_, and AUC 0-∞ are frequently used to describe patient exposure to paclitaxel.

Once an infusion has ended, plasma paclitaxel and metabolite concentrations decline over time in a triphasic manner. The first phase has a rapid decline on the order of minutes to an hour as the drug *distributes* from plasma to peripheral tissues. Subsequent phases have a slower decline where drug (i) redistributes between tissues and plasma and (ii) is *eliminated*. In an analysis exploring paclitaxel distribution from plasma to nine different organs in a rodent model, paclitaxel had substantial distribution to the liver and gut, more reduced distribution to the heart, muscle, fat, and kidney, and much less distribution in the brain (5). Furthermore, paclitaxel is primarily metabolized in the liver and excreted into bile while much less remains in plasma to be eliminated by the kidneys. In a small study tracking radiolabeled paclitaxel in 6 patients, 14% was recovered in urine and 71% in feces, most of which was a metabolite of paclitaxel, 6-α-hydroxypaclitaxel (3). Other work suggests the amount excreted in urine is even less (3%–5%) (2). These results suggest that paclitaxel quickly distributes from plasma to metabolic organs during intravenous administration. As such, it is likely that individual differences in either drug distribution or metabolism play a significant role in cellular paclitaxel exposure and the development of CIPN.

### Nonlinear pharmacokinetics

One of the interesting features of paclitaxel is that it exhibits nonlinear kinetics; with increasing dose, plasma concentrations are proportionally higher than the dose increase. For example, with a 30% increase in dose (135–175 mg/m^2^) for a 3-hour infusion, investigators have reported over a 70% increase in the maximal plasma concentration (2). Sources of paclitaxel nonlinear kinetic behavior include saturation of drug distribution among tissues in the body and drug metabolism (6). Potential mechanisms that contribute to distribution saturation include plasma and tissue protein binding of paclitaxel. In plasma, paclitaxel is highly protein bound (>90%–95%) (7) to both albumin and alpha-1-acid glycoproteins. When protein bound, it is not pharmacologically active. The remaining approximate 5%–10% unbound paclitaxel can be bound to proteins throughout peripheral tissues, transported into hepatocytes and metabolized, exert beneficial pharmacologic action inside cancer cells, or exert adverse pharmacologic action inside normal cells. Patient factors that alter protein binding such as changes in plasma protein concentrations or liver disease may influence paclitaxel distribution, clearance, and tissue penetration (3). Formulations of paclitaxel may also influence the amount of protein-bound paclitaxel. Paclitaxel is highly lipophilic and insoluble in water. Various vehicles are and have been used in intravenous preparations, including a mixture of polyethoxylated castor oil and dehydrated alcohol (Cremophor EL ®), paclitaxel bound to nanoparticles of albumin, and micelles of retinoic acid derivates that solubilize paclitaxel. In summary, nonlinear pharmacokinetics and possibly the formulation of paclitaxel introduces elements of complexity in formulating dosing regimens that are effective yet not overtly toxic to non-target tissues and cells.

### Interindividual variability in paclitaxel concentrations

As may be expected, studies measuring drug concentrations following intravenous administration have reported large differences in plasma paclitaxel concentrations and neurotoxicity between patients. In a small study of 9 patients, Huzing et al. measured plasma concentrations at 15 different times during and after a 3-hour relatively high dose infusion of paclitaxel at a rate of 250 mg/m^2^ (3). In this cohort, Cmax ranged from 2,562 to 7,686 ng/ml (3–9 micromoles/L) at the end of the infusion, and the AUC 0-∞ ranged from 15–41 µmol/l-hour. Although this study was limited by small sample size, variability in these kinetic parameters was substantial despite normalizing the dose to body surface area.

### Relationship between paclitaxel exposure and CIPN

Measuring up to 20 plasma paclitaxel samples over 48 h to estimate paclitaxel kinetic parameters is burdensome, so methods have been developed to approximate these parameters with sparse sampling and reasonable accuracy. Henningson et al. developed a sparse sampling scheme that called for only two samples in larger study cohorts (e.g., 45–50 patient subjects) to estimate pharmacokinetic parameters of interest (7).

Using this approach, Hertz et al. measured paclitaxel concentrations in 60 patients treated with paclitaxel 80 mg/m^2^ over 1 h (4), both at the end of the infusion to estimate Cmax and 16–26 h after the infusion to estimate T_C>0.05_. They also recorded patient-reported neuropathic symptoms using the CIPN8 subset of the European Organization for Research and Treatment of Cancer (EORTC) Quality of Life Questionnaire: Chemotherapy-Induced Peripheral Neuropathy (CIPN20) questionnaire. Mean and standard deviation for Cmax and T_C>0.05_ were 2,364 ± 665 ng/ml and 10.7 ± 2.3 h, respectively. To explore associations between paclitaxel exposure and CIPN, they compared Cmax and T_C>0.05_ values with CIPN8 scores, and no significant associations were found. However, in a secondary analysis, Cmax and T_C>0.05_ values were significantly associated with treatment limiting CIPN using a composite outcome measure of treatment discontinuation, delay in treatment, and dose decrease. They built a model from this relationship and estimated the paclitaxel exposure threshold that would lead to a 25% risk of treatment limiting CIPN. Their model predicted that Cmax and T_C>0.05_ values of 2,885 ng/ml and 14 h, respectively, would be associated with that risk threshold. The authors concluded that model predictions of paclitaxel exposure may be a useful tool in predicting treatment limiting CIPN and posit that a single blood draw following the first dose of paclitaxel to measure plasma concentrations and estimate exposure will predict patients at risk for treatment limiting CIPN.

In an exploratory analysis of the same cohort, Hertz et al. studied the impact of muscle mass on paclitaxel exposure (8). In patients receiving weekly 80 mg/m^2^ doses of paclitaxel, plasma paclitaxel concentrations were measured at the end of the initial infusion to estimate Cmax. Based on prior work, they used abdominal computed tomography scans to estimate muscle mass. Specifically, they used muscle mass estimates based on sagittal images through the T11 vertebrate. From this data, they built a pharmacokinetic model to predict plasma concentrations over time, and determined that using muscle mass as a covariate improved model predictions of paclitaxel plasma concentrations. Using this model, they predicted dosing adjustments to reduce the risk of exceeding a Cmax of 2,885 ng/ml. Dose adjustments consisted of expanding the infusion duration from 1 to 2 to 3 h depending on the estimate of muscle mass, with longer infusions required for patients with less muscle mass in order to limit Cmax. Although patients with elevated Cmax are more likely to have CIPN, a limitation of this work was that there was no direct exploration of a relationship between muscle mass and the presence of CIPN.

This example is one of many studies that have attempted to identify an appropriate weight or body composition scalar to guide dosing. Others have suggested that an increased body surface area puts patients at risk for developing CIPN (9). Each study presents a plausible source for elevated plasma paclitaxel concentrations. A reduced muscle mass leads to a smaller volume of distribution; a large body surface area leads to a larger dose. Future studies within the same patient cohort are required to explore the use of both measures to inform dosing and to identify measures of developing CIPN.

In summary, key features of associations between paclitaxel kinetics and CIPN include: (i) There is substantial variability in paclitaxel exposure despite dosing normalized to body surface area. This is manifested by wide variability in several kinetic parameters including Cmax, AUC 0-∞, Clearance, and T_C>0.05_. Furthermore, paclitaxel exhibits non-linear kinetics thought to be primarily a function of saturable drug distribution and metabolism. (ii) Some patients develop high plasma concentrations in response to conventional dosing while others do not, and the reason for this is not clear. High concentrations can lead to severe dose-limiting CIPN, where future doses may be postponed, reduced, or eliminated. Disruptions in dosing regimens may reduce efficacy in breast cancer treatment. Thresholds for Cmax, AUC 0-∞, Clearance, and T_C>0.05_ have been identified that are associated with increased risk of treatment limiting CIPN. (iii) Unpredictable variability has compelled investigators to seek out potential biomarkers, including biomarkers of paclitaxel metabolism, that can identify patients at higher risk of CIPN *before* starting treatment and be used to personalize treatment.

## Gene variants that influence paclitaxel metabolism and CIPN

**Figure 3:**
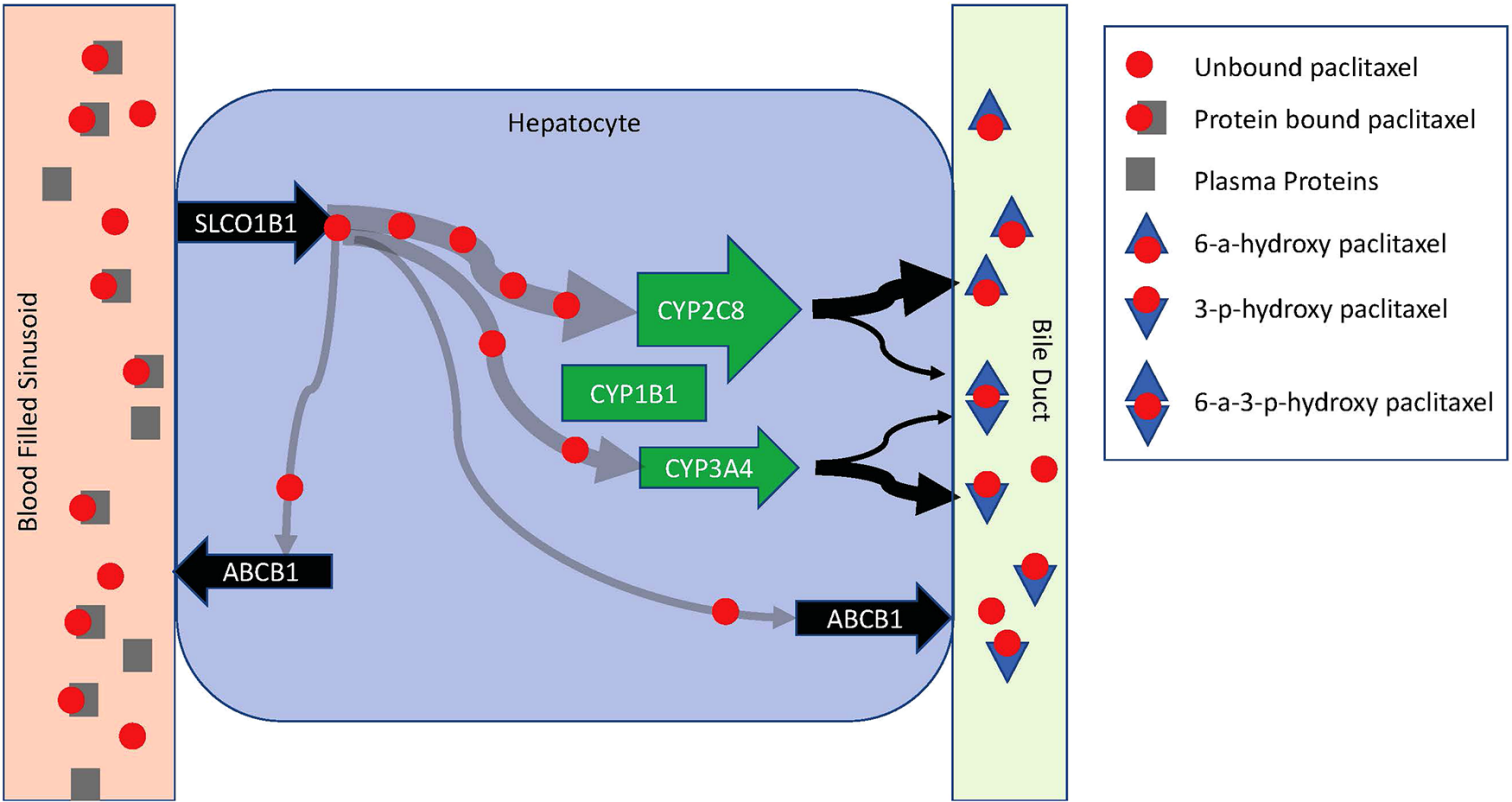
Schematic diagram of paclitaxel metabolism in a hepatocyte. Paclitaxel is introduced to a hepatocyte *via* a venous sinusoid. In the vascular compartment, it is predominantly protein-bound. Hepatocyte uptake is limited to unbound paclitaxel *via* SLCO1B1. Once in the hepatocyte, paclitaxel is primarily metabolized by CYP2C8 and CYP3A4. CYP1B1 catalyzes the metabolic activity of CYP2C8 and CYP3A4. CYP2C8 and CYP3A4 metabolize paclitaxel to 6-α-hydropaclitaxel to 3-p-hydropaclitaxel respectively. Some paclitaxel may be metabolized by both before being excreted into bile rendering 6-α-3-p-hydropaclitaxel. Metabolites are excreted into a bile duct for elimination. Free intracellular paclitaxel is moved across cell membranes out of hepatocytes back into circulation by ABCB1.

Potential mechanisms that contribute to saturable metabolism include how quickly paclitaxel can be introduced into hepatocytes and once intracellular, how quickly it can be metabolized and secreted into bile or removed from the cell back into blood (Figure 2). Enzymes in the liver-specific transmembrane transporter family, including SLCO1B1, mediate paclitaxel uptake from venous sinusoids into hepatocytes. Once inside a hepatocyte, paclitaxel is hydroxylated to hydrophilic metabolites and then excreted into the bile. Paclitaxel is primarily metabolized by the cytochrome P450 isoenzymes CYP2C8 and to a lesser extent CYP3A4. CYP1B1 also plays a role by catalyzing the metabolic activity of CYP2C8 and CYP3A4. CYP2C8 and CYP3A4 metabolize paclitaxel to 6-α-hydropaclitaxel to 3-p-hydropaclitaxel respectively. Some paclitaxel may be metabolized by both before being excreted into bile, rendering 6-α-3-p-hydropaclitaxel. In a study described above, Huzing et al. measured not only paclitaxel but also 6-α-hydropaclitaxel, 3-p-hydropaclitaxel, and 6-α-6-α-3-p-hydropaclitaxel plasma concentrations. As expected, the most prominent metabolite was 6-α-hydropaclitaxel. Unlike paclitaxel, plasma metabolite concentrations quickly diminished below the limits of detection once the treatment infusion was terminated suggesting they are quickly secreted into bile for elimination or rapidly taken up in peripheral tissues. For intracellular paclitaxel that doesn't get metabolized, membrane-associated proteins from the superfamily of ATP-binding cassette transporters, such as ABCB1, move paclitaxel across cell membranes out of hepatocytes either back into plasma or bile (Figure 3).

In the context of substantial variability in plasma paclitaxel concentrations despite dosing normalized to body size, researchers have explored associations of gene variants thought to influence paclitaxel metabolism and membrane transport with CIPN with varied results. Some reports describe associations that increase while others that decrease the likelihood of developing CIPN. Table 1 presents a compilation of selected studies that have explored associations between common variants of *CYP2C8*, *CYP3A4*, *CYP1B1*, *SLCO1B1*, and *ABCB1* with CIPN. In some of this work patient cohorts are described with variant genotypes (i.e., wild type, heterozygous, or homozygous) to allow comparison of variant prevalence within a study cohort that develops CIPN, but it is important that these are taken into consideration with the ancestry of the patient cohort as allele frequencies can differ substantially in different populations. To provide a framework for interpreting studies listed in Table 1, a key finding is presented for each study in the last column.

**Table 1:** Studies exploring associations between gene variants that influence paclitaxel pharmacokinetics and cell membrane transport with the development of CIPN.

Variant Name Author/Year	SNP ID	Cohort Size Ethnicity	Cancer Type	Study Design	Genotyping	Neurotoxicity Measurement	Relationship of Genotype to CIPN*	Paclitaxel Kinetic Analysis	Key Findings
Metabolism									
**CYP2C8**									
CYP2C8*3 Bergman et al 2011	rs10509681	93 Caucasian	Ovarian	Prospective	Wild Type: 75 (80%) Heterozygous: 19 (20%)	Not analyzed	Not analyzed	• Collected 3 plasma paclitaxel samples. • Estimated Clearance:	CYP2C8*3 was associated with an 11% lower clearance of unbound paclitaxel
CYP2C8*3 Hertz et al, 2012	rs10509681	411 European American & African American	Breast	Retrospective	Wild Type: 330 (80%) Heterozygous: 76 (18%) Homozygous: 5 (1%)	CTCAE ≥ 2	Wild Type: ∼66 (20%) developed CIPN Heterozygous: ∼25 (33%) developed CIPN Homozygous: ∼2 (40%) developed CIPN	None	CYP2C8*3 increased the risk of CIPN by 13% for Heterozygous and by 20% for Homozygous patients.
CYP2C8*3 CYP2C8*2 CYP2C8*4 Hertz et al, 2014	rs11572080 rs11572103 rs1058930	412 Caucasian & African American	Breast	Retrospective	Not provided	CTCAE ≥ 2 Version 4.0	Wild Type: ∼20% developed CIPN Heterozygous or homozygous for CYP2C8*2, *3, or *4: ∼30% developed CIPN	None	CYP2C8*2, *3, or *4 increased the risk of CIPN by 10% for Heterozygous or Homozygous patients.
CYP2C8*3 Lam et al, 2016	rs10509681	188 European	Breast	Prospective	Wild Type: 146 (81%) Heterozygous 34 (19%) Homozygous: 0 (0%)	CTCAE ≥ 2 Version 3.0	58 of 146 Wild Type (31%) developed CIPN Carriers were more likely to develop CIPN sooner, but as dose accumulation accrued, differences diminished.	None	The risk of CIPN was near equivalent at high cumulative doses of paclitaxel between Wild Type and Heterozygous or Homozygous genotypes.
CYP2C8*3 Abraham et al, 2014	rs10509681	1303 European	Breast	Prospective	Not provided	CTCAE ≥ 2 Version 2.0	No relationship between CYP2C8*3 and CIPN	None	CYP2C8*3 is of limited use as a predictor of CIPN
CYP2C8*3 CYP2C8*4 Marcath et al, 2019	rs10509681 rs1058930	58 Caucasian	Breast	Prospective	Wild Type: 40 (68%) Heterozygous 11 (19%) Homozygous: 1 (2%) Wild Type: 40 (68%) Heterozygous: 4 (7%) Homozygous: 1 (2%)	Not analyzed	Not analyzed	• Collected 3 plasma paclitaxel samples • Estimated T_C>0.05_	Patients with variants had shorter T_C>0.05_ (9.7 ± 2 h) compared to patients with no CYP2C8 variants (11 ± 2.7 h). CYPT2C8*3 & *4 may increase paclitaxel metabolism.
**CYP3A4**									
CYP3A4*22 Graan et al, 2013	rs35599367	*Exploratory Cohort:* 261 Caucasian *Validation Cohort:* 239 Caucasian	Esophageal Ovarian Breast Other	Prospective	*Exploratory Cohort* • Wild Type: 219 (84%) • Combined Heterozygous & Homozygous: 35 (16%) *Validation Cohort* • Wild Type: 211 (89%) • Combined Heterozygous & Homozygous: 26 (11%)	CTCAE ≥ 2 Version 2–4	*Exploratory Cohort* • Wild Type: 14 (6%) developed CIPN • Combined Heterozygous & Homozygous: 6 (17%) developed CIPN *Validation Cohort* • Wild Type: 20 (9%) developed CIPN • Combined Heterozygous & Homozygous: 4 (15%) developed CIPN	• Collected 3 plasma paclitaxel samples. • Estimated Cmax, Clearance, AUC 0-∞, & T_C>0.05_	Significant associations between CTCAE ≥ 2 and prolonged T_C>0.05_ & AUC 0-∞, high Cmax, and reduced clearance. No association between kinetic parameters and CYP3A4*22 variants
**CYP1B1**									
CYP1B1*3 Boora et al, 2016	rs1056836	119 Not specified	Not specified	Prospective	Not provided	CIPN20	Patients with this variant have less CIPN	None	Patients with this variant have less CIPN
*Cell Membrane Transport*									
SLCO1B1									
SLCO1B1 Abraham et al, 2014	rs3829306 rs4149013 rs4149023	1,303 European	Breast	Prospective	Not provided	CTCAE ≥ 2 Version 2.0	rs3829306 was associated with reduced CIPN rs4149013 & rs4149023 were not association with CIPN	None	rs3829306 was associated with reduced CIPN rs4149013 & rs4149023 were not association with CIPN
SLCO1B1 Di Francia et al, 2017	rs4149056	35 European	Breast, Ovarian, Genitourinary	Retrospective	Wild Type: 27 (77%) Heterozygous: 8 (23%) Homozygous: 0 (0%)	CTCAE ≥2 Version 4.0	No association with CIPN	None	No association with CIPN
SLCO1B1 Apellaniz-Ruiz et al, 2016	rs71581941	228 European	Breast, Ovarian	Retrospective	Not provided	CTCAE ≥2 Version 4.0	No association with CIPN	None	No association with CIPN
**ABCB1**									
ABCB1 Abraham et al, 2014	rs3213619	1,303 European	Breast	Prospective	Not provided	CTCAE ≥ 2 Version 2.0	rs3213619 was associated with reduced CIPN	None	rs3213619 was associated with reduced CIPN
ABCB1 C3435T Kus et al, 2016	rs1045642	219 European	Breast	Prospective	Wild Type: 30 (14%) Heterozygous:159 (73%) Homozygous: 30 (13%)	CTCAE ≥ 2 Version 4.03	Homozygous for rs3213619 was associated with reduced CIPN	None	Homozygous for rs3213619 was associated with reduced CIPN
ABCB1 C1236T ABCB1 G2677T/A ABCB1 C3435T Bergman et al 2011	rs1128503 rs2032582 rs1045642	119 Caucasian	Ovarian	Retrospective	Wild Type: 31 (26%) Heterozygous: 55 (46%) Homozygous: 26 (22%) Wild Type: 35 (29%) Heterozygous: 50 (42%) Homozygous: 29 (24%) Wild Type: 27 (23%) Heterozygous: 48 (40%) Homozygous: 39 (32%)	CTCAE ≥ 2	None of the variants were associated with CIPN	None	None of the variants were associated with CIPN
ABCB1 C1236T ABCB1 G2677T ABCB1 C3435T Tanabe et al, 2017	rs1128503 rs2032582 rs1045642	127 Japanese	Breast	Prospective	Wild Type: 20 (16%) Heterozygous: 65 (51%) Homozygous: 42 (33%) Wild Type: 53 (41%) Heterozygous: 61 (48%) Homozygous: 14 (11%) Wild Type: 48 (38%) Heterozygous: 58 (46%) Homozygous: 21 (16%)	CTCAE ≥ 2 Version 4.0	None of the variants were associated with CIPN. When adjusting for age>60 years, rs1128503 was associated with CIPN	None	None of the variants were associated with CIPN. When adjusting for age >60 years, rs1128503 was associated with CIPN

CIPN, Chemotherapy induced peripheral neuropathy; CTCAE, Common Terminology Criteria for Adverse Events; CIPN20, Quality of Life Questionnaire Chemotherapy-Induced Peripheral Neuropathy questionnaire.

A narrative is presented below highlighting key features of the studies listed in Table 1. Throughout this body of literature, a common tool in exploring associations between variants and CIPN was the use of Common Terminology Criteria for Adverse Events (CTCAE) to characterize the extent of neurotoxicity. It provides a grading scale for sensory neuropathy symptoms ranging from 1-asymptomatic, 2-moderate, 3-severe, to 4-life-threatening consequences. It is used by clinicians to estimate the severity of the neurotoxicity from paclitaxel. Unless otherwise stated, the studies presented below use CTCAE ≥ 2 as clinically significant for CIPN.

### Relationship between hepatic micro enzyme variants and CIPN

#### CYP2C8

With regards to paclitaxel neurotoxicity, perhaps the most extensively studied gene is *CYP2C8*. It is primarily expressed in hepatocytes and has several variants. Of these, CYP2C8*3 has been most rigorously studied in terms of its association with paclitaxel exposure and CIPN. Most studies report an association with CIPN, while others do not. Some examples that support and refute this association are presented below.

### Studies reporting an association between CYP2C8*3 and CIPN

Bergman et al. measured plasma paclitaxel concentrations at 3, 5–8, and 18–24 h following a 3-hour paclitaxel infusion in 93 patients to estimate paclitaxel clearance (10). Of those, 19 patients were heterozygous for CYP2C8*3, and none were homozygous. Patients heterozygous for this diplotype had an 11% decrease in clearance. Although interesting, it is important to acknowledge that kinetic model parameters based on only three samples are likely to have significant variability and lead to prediction errors of 15%–20% or more. This level of prediction variability can make distinguishing CIPN between normal and heterozygous genotypes difficult.

In an observational study, Hertz et al. reported an association between CYP2C8*3 and physician-reported CTCAE scores in 411 patients (11). Seventy-six (18%) patients developed CIPN. Heterozygous or homozygous patients and patients without this variant had a 35%–40% and 20% chance of developing CIPN respectively (*p* = 0.004). Similar findings were reported by Leskela et al. (12); patients homozygous for CYP2C8*3 were more likely to develop CIPN. Although these results are suggestive that CYP2C8*3 conveys risk of CIPN, the retrospective nature of the study and grading of neuropathic symptoms by study coordinators interpreting physician notes represent limitations and, as the authors suggest, will require prospective evaluation to validate these findings.

To that end, in a follow-up prospective study, Hertz et al. measured 564 genetic markers, including the CYP2C8*2, *3, and *4 haplotypes, in 412 paclitaxel-treated patients (13). Assuming each of these variants slowed metabolism, patients with any of these were combined into a low metabolizer group. They reported that 30% of patients in the low metabolizer group and 20% of patients with no variant developed CIPN. Although an association was identified, the difference between the low and normal metabolizing groups was statistically significant but relatively small (e.g., 10%). As the authors suggest, to effectively validate these findings would require a study that measured paclitaxel plasma concentrations in normal (wild type), carriers, & homozygous patients to confirm that CIPN was a result of elevated plasma paclitaxel concentrations and more prevalent in patients with these haplotypes.

In similar work, Lam et al. studied five genotypes in five different genes, including CYP2C8*3, in 188 patients treated with taxanes for breast cancer (14). They found that CYP2C8*3 was associated with an increased risk of CIPN. However, when comparing carriers with non-carriers using Kaplan Meier plots of cumulative paclitaxel dose vs. the proportion of patients with sensory neuropathy symptoms, both carriers and non-carriers of CYP2C8*3 had nearly identical neuropathy symptoms at higher cumulative doses of paclitaxel. In their study population, 146 patients did not have this variant, 34 were heterozygous, and none were homozygous. One hundred twenty-six patients developed grade 1, 2, or 3 peripheral neuropathy and 87 patients required a dose reduction. Although these results are suggestive of a relationship between this haplotype and CIPN, more patients developed CIPN and required dose adjustments than had the CYP2C8*3 genotype. This variant, by itself, did not have a consistent association with CIPN. Although it may be an important consideration for predicting CIPN, additional biomarkers are needed to develop tools to accurately predict who will or will not develop CIPN.

### Studies reporting no or decreased association between CYP2C8*3 and CIPN

Abraham et al. collected data from ongoing breast cancer trials and compiled a list of 50 possible genes with 73 variants thought to play a role in taxane CIPN, including CYP2C8*3 (15). In their 1,303 patient cohort, they found associations between several variants with CIPN, although CYP2C8*3 was not among them.

Analyzing blood samples from prior work suggesting CYP2C8*3 prolongs paclitaxel exposure (13), Marcath et al. measured plasma paclitaxel concentrations at two time points to estimate Cmax and T_C>0.05_ and genotyped 266 variants in 36 genes (16). They characterized *CYP2C8* genotypes in terms of normal, intermediate, and poor metabolizers based on whether patients were heterozygous or homozygous for CYP2C8*3 or CYP2C8*4. They predicted that patients with poor metabolizing *CYP2C8* haplotypes would have longer T_C>0.05_ than patients without the *CYP2C8* poor metabolizing haplotypes, but surprisingly found the opposite. Seventeen patients deemed poor metabolizers had shorter times (9.7 ± 2 h) compared to 40 patients deemed normal metabolizers (11 ± 2.7 h; *p* = 0.02). Data are expressed as mean ± standard deviation. They concluded that CYP2C8*3 may increase paclitaxel metabolism. With small group sizes and variability about the T_C>0.05_ times between patient groups of 2 to 2.7 h, the clinical implications are subtle and make it difficult to declare that the difference in T_C>0.05_ is large enough to impact clinical decisions regarding CIPN.

#### CYP3A4

Although thought to metabolize paclitaxel to a lesser extent than *CYP2C8*, researchers have studied associations of *CYP3A4* with CIPN. For example, de Graan et al. studied the development of neuropathic symptoms in patients being treated with paclitaxel for a variety of cancers in men and women (17). Their experimental design was particularly noteworthy—they explored associations between drug concentrations and exposure with CIPN as well as associations between genotypes and CIPN. They enrolled 261 patients in an exploratory cohort and 239 patients in a validation cohort with a variety of tumor types. They measured paclitaxel plasma concentrations and estimated pharmacokinetic parameters Cmax, AUC 0-∞, Clearance, and T_C>0.05_. using sparse plasma sampling in a large patient cohort as described above by Hennigsson et al. (7). As others have reported, they confirmed that increased paclitaxel exposure was associated with CIPN. They genotyped patients for four drug metabolizing enzymes, including CYP3A4*22. In the exploratory cohort, female carriers of CYP3A4*22 were at increased risk of developing CIPN, but this was not observed in male patients. In the validation cohort, both female and male carriers were at risk. Of note, there were no patients with a homozygous genotype in either cohort, which is not surprising given that the *22 allele has a frequency of approximately 5% (18–20). They found no associations between CYP3A4*22 and the kinetic parameters studied. The authors concluded that female carriers of CYP3A4*22 may be at increased risk of developing CIPN and that it may be useful in personalizing therapy. Other work has put forth similar findings that unique *CYP3A4* variants may have an association with paclitaxel dose limiting CIPN (21). Given that a premise of this variant is that it would disrupt metabolism and prolong paclitaxel exposure, it is interesting that there was no association between CYP3A4*22 and Cmax, AUC 0-∞, Clearance, or T_C>0.05_.

Given the relatively rare frequency of the CYP3A4*22 genotype it is unlikely to explain CIPN in the majority of patients. To put the results of de Graan et al. into perspective, it is useful to compare the prevalence of CIPN across genotypes. In their exploratory cohort, 14 out of the 219 patients with a normal metabolizing genotype (6%) developed CIPN, and 6 out of 35 patients with heterozygous genotypes (17%) developed CIPN. Similarly, in their validation cohort, 20 out of the 211 patients with the normal metabolizing genotype (9%) developed CIPN and 4 out of 26 patients with heterozygous genotypes (15%) developed CIPN. It is important to recognize that between 6% and 9% of patients without this variant developed CIPN. Thus, although CYP3A4*22 conveys a modest increase in risk of developing CIPN, it is clear that other mechanisms independent of this variant also contribute to CIPN.

#### CYP1B1

CYP1B1 catalyzes reactions involved in metabolizing several drugs including the metabolism of paclitaxel, and is involved in other functions including the synthesis of cholesterol, lipids, and steroids. Researchers have found that variants of this gene may have a protective effect in reducing CIPN, other chemotherapy related toxicities such as taxane hyper-sensitives (e.g., dyspnea, flushing, rash, etc.) (22), and improve survival (23). Although not as well studied, prior work suggests that it has a role in mitigating paclitaxel CIPN. Boora et al. studied 22 variants within 16 genes in 119 patients undergoing paclitaxel treatment for breast cancer and explored their association with CIPN (24). They found that CYP1B1*3 demonstrated a protective effect diminishing the risk of CIPN and suggested that it may confer increased survival in patients with stage II or IV breast cancer as has been previously suggested by others (25). A limitation of this work was that detailed information about allele frequency in this cohort was not provided, making it difficult to understand how well the variant provided a protective effect.

### Relationship between cell membrane transport protein variants and CIPN

Variants in genes that code for proteins involved in paclitaxel transport into and out of cells may contribute to variations in internal drug exposure and risk of developing CIPN. Some of these genes include *SLCO1B1* and *ABCB1*. *SLCO1B1*, one gene in a family of genes that codes for membrane transport proteins, transports primarily organic anions, such as paclitaxel, into cells. *ABCB1* is a member of the ATP-binding cassette subfamily, a permeability glycoprotein that codes for an ATP-dependent membrane transport protein. This protein excretes many foreign substances, including paclitaxel, out of cells. Several studies have evaluated associations between variants of these genes and CIPN.

#### SLCO1B1

Several variants for *SLCO1B1* have been identified, but for the most part, their association with CIPN has not been clearly elucidated. Variants may either slow or accelerate hepatocyte uptake. For example, in the study described above, Abrahams et al. (15) studied associations between development of CIPN and variants of *SLCO1B1* include rs3829306, rs4149013, and rs4149023. Although they found a borderline statistically significant association between the T allele in rs3829306 and CIPN, the strength of the association was weak.

In 76 patients diagnosed with breast, ovarian, or genitourinary cancer treated with paclitaxel or docetaxel, Di Francia et al. studied a set of 7 genes with variants thought to have associations with CIPN (26). They investigated the association of heterozygous and homozygous genotypes of the *SLCO1B1* variant rs4149056 with CIPN and reported an odds ratio of nearly 1, concluding there was no relationship between this variant and CIPN.

Apellaniz-Ruiz conducted a retrospective analysis exploring associations between 39 candidate genes with CIPN in patients with breast and ovarian cancer treated with paclitaxel (21). They focused on genes associated with neuronal injury repair, paclitaxel metabolism and transport, and Charcot-Marie-Tooth hereditary neuropathies. Using targeted sequencing, they searched for variants in *SLCO1B1* among other genes in a cohort of 228 patients: 131 with grade 2–3 neuropathy and 97 with grade 0–1 neuropathy. They detected 277 variants among the 39 candidate genes. Most were rare, but 86 were frequently occurring (MAF > 5%). They identified 4 variants for SLCO1B1 and 5 variants for SLCO1B3, none of which had an association with CIPN. They validated these negative findings in a separate cohort of 202 patients.

In a systematic review and meta-analysis of the pharmacogenetics of taxane-produced CIPN in breast cancer patients, Guijosa et al. evaluated previously published associations in 262 variants across 121 genes in 42 studies with 19,431 patients (27). They leveraged their systematic review to explore potential associations that were not otherwise detectable in single studies. They focused on four variants of *SLCO1B1*, rs4149056, rs11045819, rs11045819, and rs34671512. They found that the overall variant effect was significant for a *decrease* in neurotoxicity from paclitaxel.

#### ABCB1

Available studies exploring associations between variants of *ABCB1* and sensory neuropathy are sparse and similar to other gene variants, providing conflicting results. A few of the available studies are described below. Abraham et al., in their previously described analysis found the variant rs3213619 of *ABCB1* to have a significant association with CIPN (15). They concluded that this variant among others may contribute to CIPN but because of substantial interindividual variability, further studies are warranted to better elucidate genes, variants, and pathways that contribute to CIPN.

In a retrospective observational study of 219 patients treated with paclitaxel or docetaxel for breast cancer, Kus et al. collected DNA samples and conducted a candidate gene analysis of variants in 8 genes thought to play a role in developing CIPN (28). They explored associations between the *ABCB1* variant rs1045642 and found patients homozygous for the T allele had *increased* risk for CIPN [OR: 2.76 (1.17–6.49); *p* < .05].

In a retrospective observational candidate gene study in 119 patients treated with paclitaxel in combination with carboplatin for ovarian cancer, Bergmann et al. genotyped tumor tissues for 22 variants in 10 genes. In particular, they studied three variants in *ABCB1*, rs1128503, rs2032582, and rs1045642 (29). They explored associations between these variants and survival, neuropathy, and neutropenia, and found no statistically significant associations with any of these outcomes.

In an observational study of 92 Egyptian patients treated with paclitaxel for breast cancer, Abdelfattah et al. explored associations between selected variants of *ABCB1* and CIPN (9). They studied two variants of *ABCB1*, rs1128503, and rs1045642. Patients homozygous for rs1128503 had a significant association with CIPN along with patients that had a large body surface area and those with diabetes. They concluded that the presence of this variant, large body surface area, and/or history of diabetes are reasonable predictors of CIPN and should be considered to personalize paclitaxel dosing to minimize or prevent CIPN.

In an observational study of 127 Japanese women being treated with paclitaxel for breast cancer, Tanabe et al. collected blood samples to explore associations between selected variants in *ABCB1* among other genes and CIPN (30). In their analysis, they found a marginally significant association with the TT genotype for *ABCB1* rs1045642 with CIPN, which became more significant upon adjusting for age >60 years.

To summarize, key findings of studies exploring associations between gene variants involved in paclitaxel metabolism and membrane transport and CIPN include an uncertain association between CYP2C8*3 and CIPN, as well as a potentially increased risk of CIPN in patients with CYP3A4*22. Whether variants in *ABCB1* or *SLCO1B1* contribute to or reduce CIPN from paclitaxel remains difficult to determine. Many of these studies limited because they include small numbers of patients, the patient cohorts are heterogeneous, and the study designs fail to account for multiple comparisons.

Assuming variants in these transporter genes modify risk of neuropathic symptoms from paclitaxel, it is interesting to consider potential mechanisms. One possibility is that some genotypes result in increased transport of paclitaxel into hepatocytes allowing for more efficient metabolism. This would correspond to lower plasma concentrations, less neurotoxicity, and perhaps less effectiveness as a chemotherapeutic agent. However, even if this or other PK/PD mechanisms influence risk of CIPN, the variation explained is incomplete, necessitating the discovery of additional mechanisms predisposing patients to CIPN.

Importantly, for many of the pharmacogenomic haplotypes investigated in these studies, relatively few patients have rapid or poor metabolizing genotypes and many of those that developed CIPN were predicted to metabolize paclitaxel normally. Therefore, the utility of variants involved in the direct metabolism and transport by themselves to predict the risk of CIPN is likely of limited use, and additional predictors will be needed.

## Future directions

From the body of work described in this narrative review, it is evident that some patients without any of these gene variants can develop CIPN, necessitating identification of additional predictors in order to develop a clinical tool that will accurately predict CIPN. The heterogenous associations between a genetic variant and CIPN may be due to study design factors (e.g., ethnicity, cancer type, phenotype definitions, treatment regimens) or due to other unmeasured molecular factors that interact with these genetic variants. Notably the sample sizes for many of the prior pharmacokinetic studies are small and larger studies are needed to ensure sufficient statistical power to observe potential impacts on CIPN. Furthermore, the impact of genetic variation on protein function is only part of the story, the expression of some pharmacogenomics genes (e.g., CYP3A4) are inducible by concomitant medications, environmental exposures, or other factors and may also play a role in paclitaxel metabolism. Leveraging available techniques such as RNA sequencing, DNA methylation assessment, and inflammatory protein quantification, of samples before, during, and after treatment may provide insights into other mechanisms that could help explain additional variation in CIPN. Another interesting possibility is that in addition to potential enzymatic efficiency due to genetic variation, changes in enzyme abundance due to modified gene or protein expression may impact CIPN, and may be modified over time in response to paclitaxel treatment. To that end, mapping the onset and duration of CIPN to other mechanisms may be useful.

For example, measuring differential expression of micro-RNAs (miRNAs), mRNAs, and DNA methylation, all of which have not only been used to predict breast cancer tumor responses to paclitaxel (31) but to evaluate regulation of enzymes responsible for drug metabolism (32), may prove useful in characterizing how variants influence paclitaxel metabolism. Some examples include: (i) miRNAs miR-452–5p and miR-224-5p downregulate *CYP2C8* and *CYP3A4* (33) and (ii) Methylated cytosine followed by guanine nucleotides in linear sequence, referred to as CG or CpG islands, in promoter regions of *CYP3A4* suppress its expression. Methylation inhibitors increase expression of both *CYP3A4* (34).

A more comprehensive approach to exploring the variability of paclitaxel kinetics include: (i) measuring expression of miRNAs and methylation of CG islands in DNA that influence transcription of metabolic genes, (ii) consideration of these epigenetic mechanisms in the expression of metabolic gene variants, and (iii) exploration of associations between variant expression, plasma paclitaxel concentrations, and CIPN. Beyond single nucleotide polymorphisms, inclusion of metabolic, clinical, and demographic data may provide a more in-depth exploration of contributions to CIPN. A multi-omic approach with data from the genome, epigenome, transcriptome, and metabolome, offers an opportunity to explore associations between promising combinations. For paclitaxel kinetics, a multi-omic approach would include an exploration of gene variants associated with paclitaxel metabolism, their genotype presentation, miRNAs and DNA methylation that influence their transcription, and their mRNA expression in combination with covariates, such as drug administration time, dose, age, muscle mass, smoking history, and comorbidities of interest such as diabetes or disorders associated with neuropathy. An important assumption is that several “omic-demographic” profiles associated with either an increased or decreased risk of developing CIPN may exist.

## Discussion

Given the difficulty in predicting which patients will develop CIPN, investigators have searched for biomarkers that will identify patients who are likely to have elevated paclitaxel concentrations. It has been well established that paclitaxel exhibits (i) nonlinear kinetics that are a function of saturable distribution and metabolism and (ii) substantial variability in paclitaxel concentrations despite normalizing dose to body surface area. A consistent finding was that kinetic variables used to describe high plasma concentrations of paclitaxel had a strong association with CIPN. So much so that measuring paclitaxel concentrations at the end of an initial treatment may be a useful predictor of CIPN development although clinical validation is warranted.

Several studies have explored factors that influence paclitaxel metabolism and distribution. Most published work has focused on enzymes involved in either paclitaxel metabolism or membrane transport. Variants of several genes involved in these mechanisms show promise, although, none have emerged, by themselves, as a consistent predictive biomarker of CIPN. Some results for selected gene variants, when considered as a cumulative body of work, are difficult to interpret because of conflicting results.

Gene variants, by themselves, may not be sufficient as robust predictors of CIPN. Patients have heterogenous genetic, clinical, and demographic presentations. One single biomarker will likely not have broad enough coverage over a general population as a predictor of CIPN. To be more comprehensive, collections of biomarkers combined as biomarker signatures across several data domains are needed. Leveraging machine learning techniques, future work should next explore multiple -omic data domains in combination with demographic and clinical profiles to more accurately identify patients at risk for CIPN. These advances will allow patients to receive continuous therapy against a devastating disease as well as improve their overall quality of life during and after treatment.
